# Editorial: Microbial stress mitigation and crop improvement using multiomics holistic approach

**DOI:** 10.3389/fmicb.2025.1682186

**Published:** 2025-09-24

**Authors:** Renu S., Prem Lal Kashyap, Khan Mohd. Sarim

**Affiliations:** ^1^Indian Council of Agricultural Research, Krishi Bhawan, New Delhi, India; ^2^ICAR-Indian Institute of Wheat and Barley Research, Karnal, Haryana, India; ^3^Institut Rudjer Bošković, Zagreb, Croatia

**Keywords:** abiotic and biotic stress, crop improvement, multiomics technologies, mycorrhizal fungi (MF), nanotechnology

In the face of growing global challenges such as soil degradation, salinization, climate variability, and increasing biotic stress due to diseases and pests, achieving sustainable crop production requires innovative and ecologically sound approaches (Monjezi et al., 2025; Kashyap et al., 2025). Recent breakthroughs in plant–microbe interactions, soil health, and multiomics technologies (e.g., genomics, transcriptomics, proteomics, and metabolomics) along with nanotechnology have opened new avenues for transformative solutions in agriculture and allied sectors, with a strong focus on stress mitigation and crop improvement through microbial resources at molecular and system levels (Srikanth et al., 2025; Sahoo et al., 2025). In light of these advancements, our Research Topic, “*Microbial stress mitigation and crop improvement using multiomics holistic approach,”* presents a timely collection of six articles offering a comprehensive understanding of how microbial interventions and multiomics tools can support climate-resilient and sustainable agriculture. This compilation explores novel strategies and applications, including the use of mycorrhizal fungi for plant growth promotion and disease management, mechanisms underlying microbial community dynamics under saline environments, and the role of nanomaterials in crop improvement.

Umer et al. provided a comprehensive review on the role of arbuscular mycorrhizal fungi (AMF) in promoting plant growth and managing diseases. The review presents a systematic understanding of how AMF enhances plant growth and disease resistance, contributing to sustainable crop production. AMF improves nutrient and water uptake through extensive extraradical hyphal networks, thereby boosting host plant performance. Additionally, AMF colonization modulates phytohormone signaling pathways, primes systemic resistance, and upregulates defense-related genes. This leads to increased biosynthesis of secondary metabolites, strengthening of cell walls, and activation of antioxidant enzymes. AMF also enhances the biocontrol efficacy of rhizospheric microbes through synergistic interactions. The role of AMF in establishing disease-suppressive soils via multiple mechanisms is discussed. Omics studies have revealed specific interactions between AMF and hosts, as well as their functional dynamics. For successful field application and integration into resilient and sustainable agriculture, further research is needed on tripartite interactions, formulation technologies, and long-term ecosystem implications.

Shao et al. explored the mechanisms shaping the sorghum rhizosphere microbial community under a sorghum–peanut intercropping system in salt stress conditions. Using four different treatments, the study identified significant changes in soil metabolites between normal and salt-stressed conditions, demonstrating how intercropping alters microbial community structure by influencing soil sugar metabolism. Under salt stress, sucrose and fructose levels were significantly reduced, suggesting their consumption by plants and microbes to mitigate stress. Additionally, salt-stressed intercropped conditions promoted beneficial microbes such as *Rhodanobacter* and *Rhizopus*. A strong correlation between these microbial taxa and sugar metabolites indicates that shifts in metabolite profiles directly influence microbial composition and function. This study highlights the critical role of plant–metabolite–microbe interactions in alleviating salinity stress, offering a promising strategy for sustainable agriculture.

Yingtao et al. investigated the defense mechanisms of *Psammosilene tunicoides*, a medicinal plant known for its anti-inflammatory, antioxidant, and immunomodulatory properties, which is susceptible to root rot disease. Utilizing integrated omics approaches and culturomics, they analyzed both healthy and diseased roots and rhizosphere soils. Transcriptomic, metabolomic, and microbial profiling revealed that flavonoid and triterpenoid metabolism are central to the plant's resistance against root rot. Genes involved in the biosynthesis of these compounds were significantly upregulated in diseased plants. In response to external stress, *P. tunicoides* roots secrete specific secondary metabolites that resist pathogen invasion and enrich beneficial microbial communities. These findings offer valuable insights for ecological cultivation and disease management of *P. tunicoides*, with potential applications in rhizosphere microbiome engineering.

Hernández-García et al. assessed how different fertilization strategies influence soil microbial communities and crop performance. Their study compared conventional fertilization with biofertilization using a synthetic microbial consortium (SynCom) derived from teosinte-associated microbes. Results revealed distinct shifts in soil microbiota under both treatments. Biofertilization, especially via drone application, enhanced populations of beneficial microbes while reducing potentially harmful taxa. Specifically, Enterobacteriaceae genera were reduced, whereas beneficial genera such as *Bacillus, Pantoea*, and *Serratia* were enriched. Drone-assisted delivery promoted complex microbial networks, improving soil resilience and crop growth. These findings highlight the importance of microbial dynamics in biofertilization, and its role in sustainable agriculture.

An et al. explored how continuous vs. rotational cropping influences the soybean root bacterial microbiome. Using 16S rDNA sequencing and soil metabolomics, they compared the effects of continuous soybean cropping and corn–soybean rotation. Continuous cropping led to reduced bacterial diversity and altered community composition, whereas rotational cropping improved plant growth, yield, and soil nutrient content. Metabolite analysis also showed that continuous cropping and rotation differentially influenced metabolite profiles, reducing sucrose levels and overall metabolic activity. Rotation also altered bacterial translocation between the rhizosphere and endosphere. The strong correlation between soil metabolites and microbial community composition emphasizes the key role of root-associated microbes and metabolites in improving crop health and productivity.

Sodhi et al. reviewed the application of nanomaterials in enhancing plant growth and managing both biotic and abiotic stresses, such as soil salinity, extreme climates, and phytopathogen attacks—all major threats to agricultural productivity. The review discusses nanomaterial synthesis from various biological sources, including microbes, plants, and algae. Nanomaterials can improve nutrient absorption, water retention, and targeted delivery of active compounds, while also strengthening plant antioxidant defenses and photosynthetic efficiency. They offer protection against pathogens and pests and foster beneficial plant–microbe interactions. The authors highlight the importance of developing safe, biodegradable nanomaterials and advanced nanotechnologies for effectively and sustainably addressing agricultural stress.

In conclusion, the integration of microbial research with multiomics platforms is transforming our approach to crop improvement. By uncovering the genomic and functional traits of beneficial microbes at the community and system level, these research efforts clearly demonstrate that microbial interventions, combined with multiomics tools, can foster climate-resilient and sustainable agriculture. Together, they pave the way for robust, climate-adaptive farming systems that are essential for ensuring global food security amid growing environmental challenges.
